# Editorial: Immunological aspects of vaccine safety

**DOI:** 10.3389/fimmu.2023.1212148

**Published:** 2023-08-18

**Authors:** Michael Vajdy, Barbara A. Rath, Kawsar R. Talaat

**Affiliations:** ^1^ Pathomune LLC, Sacramento, CA, United States; ^2^ Vaccine Safety Initiative, Berlin, Germany; ^3^ Center for Immunization Research and the Institute for Vaccine Safety, Johns Hopkins Bloomberg School of Public Health, Baltimore, MD, United States

**Keywords:** vaccines, safety, toxicity, pharmacovigilance, immunology, SARS-CoV-2

Vaccines to prevent infectious diseases are usually administered to healthy individuals. Therefore, the benefits must outweigh the risks, i.e. they must prove relatively safe. The safety of vaccines is determined by a number of factors, all of which contribute to the benefit-risk ratio, which is the basis of licensing decisions taken by regulatory agencies.

The prevailing current notion in immunology is that vaccination should induce strong innate pro-inflammatory responses. The length of time a vaccine remains *in situ* around the injection site, before it is distributed systemically, is another important factor that directly correlates with the extent of the local and systemic immunity and adverse events following immunization (AEFI).

Possible contributors to AEFI are excessive or prolonged innate pro-inflammatory responses (1, 2). The induction of systemic IL-1β may lead to fever, a well-known systemic AEFI with several licensed vaccines. Moreover, overt neutrophil infiltration at the injection site may result in local inflammation and pain (1, 2).

National and international regulatory agencies have developed priority pathways for pandemic pathogens allowing fast-tracked vaccine and drug development. Among the initial candidates for COVID-19 vaccines were mRNA/lipid nanoparticle and adenovirus-based viral vector vaccines. As the pandemic is ending soon, and these successful novel technologies are applied to vaccines against other pathogens, questions must be asked and answered. More work remains to be done to explore the opportunities and limitations of mRNA vaccine technology in the future. Large funding bodies should step up and encourage public/private partnerships and national and international research programs to undertake detailed studies on the nano- micro-and macrolevel, as well as head-to-head comparisons across vaccine platforms and technologies.

Administration of a SARS-CoV-2 mRNA-based vaccine has been shown to result in a decrease in reticulocytes and platelet counts (3) and low platelet counts, also known as thrombocytopenia, can also be associated with thrombosis (4). This may explain in part why, in rare instances, mRNA-based SAR-CoV-2 vaccinations could increase risk of thrombosis in humans, albeit at the rate of one in 100,000-1,000,000. Bilotta et al. performed a systematic review of thrombosis cases after two mRNA- and one adenovirus-based vaccine against SARS-CoV-2. They found that in 3 reported cases, thrombosis occurred within 3 days following immunization with mRNA vaccines, accompanied by low platelet counts and petechiae/purpura. Thrombosis has also been observed in rare instances following immunization with an adenovirus-based vaccine; in 58 affected individuals, symptoms had appeared by day 9, and included headache and low platelet count (Bilotta et al.).

Another critical component is ongoing pharmacovigilance of existing and future vaccines such as the Vaccine Adverse Event Reporting System (VAERS). VAERS is a signal detection safety system run by the FDA and CDC that passively collects reports from the public, physicians, and manufacturers. It cannot ascribe causality to a specific vaccine nor provide a denominator of the number of vaccines received with no AEFI reported. Even though the system is very powerful due to large numbers, no comparison group exists in VAERS to assess increases over background rates. Following review of reported adverse events in VAERS through December 2020, Chen et al. found that mRNA-based vaccines in the vast majority of cases have been safe. Their analysis occurred after 1.8 million doses were administered; in examining VAERS, 0.2% reported an AEFI. Known AEFI that occurred in up to 50% of vaccinees included both local, such as injection site pain and systemic, i.e. fatigue, pain, chills, vomiting, nausea, headache, myalgia, arthralgia (Chen et al.).

Besides passive surveillance, vaccine developers should screen for the pathogenesis and biological link of any AEFI detected while studying in detail safety signals observed during pre-clinical studies. Preclinical studies rarely provide toxicity data from animal models and *in vitro*, but they may provide important clues for further development. For example, Banihashemi et al. and Tran et al. performed histopathological analyses in various tissues, and single dose and repeat dose toxicity of protein-based combined intra-muscular (IM) and intra-nasal or IM vaccinations, respectively, using adjuvanted recombinant spike protein of SARS-CoV-2, in several mammalian species. Another promising approach will be the identification of vaccine safety biomarkers predicting who may be at risk of developing AEFI. Sasaki et al. measured various genetic biomarkers in the lungs following intra-nasal vaccinations, with an inactivated trivalent whole-virion influenza vaccine containing one of several adjuvants (either Aluminum hydroxide, squalene oil-in-water, murine stimulator of interferon genes (STING) ligand DMXaa, toll-like receptor 1/2 (TLR1/2) agonist Pam3CSK4, TLR3 agonist Poly I:C, TLR7/8 agonist R848, Silicon dioxide nanopowder (NanoSiO_2_), or the TLR9 agonist CpG K3). One of their important findings was that increased expression of the Ifi47, C2, and Csf1 genes, positively correlated with leukopenic toxicity (Sasaki et al.). Ifi47 influences the TNF family (Sasaki et al.), which is involved in hematopoiesis (5), C2 is involved in coagulation and complement pathways, and Csf1 is involved in both the hematopoietic and TNF pathways (Sasaki et al.).

Such studies ideally should also include measuring pro-inflammatory cytokines and chemokines, liver enzymes and acute phase inflammatory liver biomarkers in blood, as well as inflammatory molecules in easily accessible mucosal secretions such as saliva and nasal swabs, complete blood chemistry and platelet and differential WBC counts as well. Wang et al. studied immune signatures after vaccination in 50 individuals vaccinated with the inactivated SARS-CoV-2 vaccine, CoronaVac. Utilizing a variety of methodologies, they found that humoral and complement compartment expression and activation were greater than other immune-associated pathways with a lack of upregulation of inflammatory cytokines and acute phase proteins seen in severe COVID infection (Wang et al.). Another example is examining innate and adaptive immune profiles when an MVA-vectored HIV vaccine (MVA-B) is administered either transcutaneously or IM (Sanchez et al.). The investigators found distinct AEFI triggered by different immune mechanisms; the transcutaneous route triggered a CD8-mediated response without antibody development and with less inflammatory cytokines and reactogenicity and the intramuscular route induced both strong antibody development, some CD8 activation, and higher IL-6 levels. The volunteers who received the vaccine via the intramuscular route had more local and systemic symptoms such as fatigue and myalgias as well as pain and tenderness at the injection site than did the volunteers who received the identical vaccine via the transcutaneous route.

Another potential aspect of vaccine safety is vaccine-induced Antibody Dependent Enhancement (ADE) following infection or disease. Ricke suggested that SARS-CoV-2 related vaccines or infections might elicit mechanisms of ADE, *i.e*. the binding of non-neutralizing IgG antibodies in the vaccinee to FCγ1 receptors on various immunocytes or mast cells. However, results of clinical trials and post-marketing surveillance undertaken thus far do not support that notion.

Comprehensive vaccine safety studies in the future should involve children and elderly people, especially those above 75 years of age to gather more information in safety and efficacy at the extremes of age. Yang et al. support this notion by calling for more systematic clinical and safety trials in specific vulnerable population groups such as pregnant women and newborns.

Laboratory analysis in all age groups should include absolute lymphocyte numbers and subtypes. These kinds of studies may yield surprising results: tetanus/diphtheria toxoid vaccination in children with solid tumors resulted in fluctuations in numbers of various lymphocyte populations (Kostinov et al.).

At the same time, Vaccine Pharmacovigilance needs to be strengthened with regular analyses published, including layperson summaries available to the general public. For example, once any vaccine is licensed in healthy adults, and the vaccine is provided to the public, vaccine pharmacovigilance continues using VAERS and similar databases; however prospective studies remain necessary. The publications in this Research Topic highlight the importance of continued efforts at exploring mechanisms of vaccine safety, from immunological as well as toxicological aspects.

## Author contributions

MV wrote the majority of the Editorial, and KT and BR contributed by adding statements about the papers they edited as well as other overall editing. All authors contributed to the article and approved the submitted version.

## References

[1] Kang S-M Compans RW. Host responses from innate to adaptive immunity after vaccination: molecular and cellular events. Mol Cells (2009) 27(1):5–14. doi: 10.1007/s10059-009-0015-1 19214429 PMC6280669

[2] Herve C Laupeze B Del Giudice G Didierlaurent AM DaSilva FT. The how’s and what’s of vaccine reactogenicity. NPJ Vaccines (2019) 4:39. doi: 10.1038/s41541-019-0132-6 31583123 PMC6760227

[3] Rohde CM Lindemann C Giovanelli M Sellers RS Diekmann J Choudhary S. Toxicological assessments of a pandemic COVID-19 vaccine—Demonstrating the suitability of a platform approach for mRNA vaccines. Vaccines (2023) 11:417. doi: 10.3390/vaccines11020417 36851293 PMC9965811

[4] Gogoleva VS Atretkhany K-SN Dygay AP Yurakova TR Marina S Drutskaya MS. Current perspectives on the role of TNF in hematopoiesis using mice with humanization of TNF/LT system. Front Immunol (2021) 12:661900. doi: 10.3389/fimmu.2021.661900 34054827 PMC8155636

[5] Ashworth I Thielemans L Chevassut T. Thrombocytopenia: the good, the bad and the ugly. Clin Med (2022) 22(3):214–7. AXB. doi: 10.7861/clinmed.2022-0146 PMC9135082 35584828

